# Dynamic Assessment of Local Abdominal Tissue Concentrations of Cisplatin During a HIPEC Procedure: Insights from a Porcine Model

**DOI:** 10.1245/s10434-025-17000-w

**Published:** 2025-02-12

**Authors:** Christina Harlev, Mats Bue, Elisabeth Krogsgaard Petersen, Andrea René Jørgensen, Bo Martin Bibby, Pelle Hanberg, Anne Vibeke Schmedes, Lone Kjeld Petersen, Maiken Stilling

**Affiliations:** 1 Department of Clinical Medicine, Aarhus University, Aarhus N, Denmark; 2 Department of Orthopaedic Surgery, Aarhus Denmark Microdialysis Research (ADMIRE), Orthopaedic Research Laboratory, Aarhus University Hospital, Aarhus, Denmark; 3 Department of Orthopaedic Surgery, Aarhus University Hospital, Aarhus, Denmark; 4 Department of Biostatistics, Aarhus University, Aarhus, Denmark; 5 Department of Biochemistry and Immunology, University Hospital of Southern Jutland, Vejle, Denmark; 6 Department of Gynecology and Obstetrics, Odense University Hospital, Odense, Denmark; 7 Department of Clinical Research, University of Southern Denmark, Odense, Denmark

**Keywords:** HIPEC, Ovarian cancer, Cisplatin, Pharmacokinetics, Microdialysis, Porcine model

## Abstract

**Background:**

This study aimed to establish a feasible large porcine model for dynamic assessment of cisplatin concentrations in carcinomatosis-relevant abdominal tissues using microdialysis during and after HIPEC combined with cytoreductive surgery.

**Methods:**

In total, eight pigs underwent open abdominal cytoreductive surgery followed by HIPEC. Microdialysis was employed for dynamic cisplatin concentration sampling in abdominal organs and tissue. Cisplatin dialysate concentrations were analyzed using the UPLC-MS/MS method. STATA (version 18.0) was used to perform a two-compartment model with a zero-order distribution to analyze pharmacokinetic parameters.

**Results:**

Detectable cisplatin concentrations in the evaluated target tissues persisted for at least 6 h post-HIPEC. Higher concentrations were found in superficial tissues; however, the difference was not statistically significant. The cisplatin concentrations were comparable for the stomach, rectum, and liver but higher in the peritoneal lining of the abdominal wall, with the lowest median average peak concentration (*C*_max_) in the rectum (0.50 µg/mL) and the highest median *C*_max_ in the peritoneum (2.80 µg/mL). No statistically significant differences in cisplatin area under the curve from time zero to the time of the last sample collection (AUC_0–last_) were found between any of the abdominal compartments except the peritoneal lining of the abdominal wall, which was significantly higher compared with most of the other abdominal tissues {smallest difference; peritoneum 1/liver 2; 1.96 [95% confidence interval (CI) 0.90; 4.26, *P* = 0.09] and largest difference; peritoneum 3/rectum profound; 4.60 [95% CI 1.94; 10.90, *P* = 0.001]}.

**Conclusions:**

Our investigation revealed comparable cisplatin concentrations across abdominal organ surfaces, except higher concentrations in the peritoneal lining of the abdominal wall than in the stomach, rectum, and liver. This model holds promise for future research into HIPEC interventions and anticancer effectiveness.

**Supplementary Information:**

The online version contains supplementary material available at 10.1245/s10434-025-17000-w.

Peritoneal carcinomatosis in patients with ovarian cancer is associated with poor prognosis and rapid disease recurrence despite extensive macroradical cytoreductive surgery and systemic antineoplastic treatment.^1–5^ Hyperthermic intraperitoneal chemotherapy (HIPEC) is an intraoperative procedure that delivers chemotherapy directly into the abdominal cavity after cytoreductive surgery. A significant, 12-month prolongation of overall survival has been reported in patients with advanced stage III ovarian cancer in the OVIHIPEC-1 trial in which cytoreductive surgery was combined with HIPEC in patients undergoing interval cytoreductive surgery compared with patients who received cytoreductive surgery alone.^6^ In Europe, HIPEC is increasingly used in combination with cytoreductive surgery in the treatment of peritoneal carcinomatosis. However, the core elements of optimal HIPEC therapy, i.e., dose and administration regimens, treatment time, and the role of temperature during treatment, remain largely unexplored.^7^ This means that a protocol for obtaining ideal cytotoxic chemotherapy exposure in carcinomatosis-relevant tissues remains to be established.

Cisplatin is a cell-cycle nonspecific alkylating agent believed to act synergistically with hyperthermia^1^ and is, therefore, often used in HIPEC. Research on local abdominal tissue concentrations has primarily involved biopsies in small animal models following intraabdominal injections, while plasma cisplatin levels during HIPEC are well described.^8–10^ The translational potential of these models is limited and lacks temporal evaluations.^11,12^ We have previously developed a similar porcine model for sampling and monitoring the pharmacokinetic profile of carboplatin administered through HIPEC after cytoreductive surgery.^13^ This experience has provided valuable insights into the practical and methodological aspects of evaluating chemotherapy distribution in abdominal tissues.

Microdialysis is a well-established pharmacokinetic sampling method for the dynamic evaluation of extracellular fluid concentrations of different drugs, including antineoplastic agents, simultaneously from various tissues.^14–16^ Microdialysis is a valuable tool for scrutinizing the pharmacokinetic profile of cisplatin during HIPEC, providing a meticulous and real-time analysis of target site drug concentrations. Microdialysis only samples the unbound drug fraction, which is directly correlated to therapeutic efficacy, presumably because the unbound molecules are those capable of crossing cell membranes, interacting with specific receptors, and triggering the desired pharmacological response.^17^

This study aimed to evaluate the feasibility of a novel large porcine model to dynamically assess free cisplatin concentrations in tissues from various abdominal organs (stomach, the peritoneal lining of the abdominal wall, rectum, and liver) using microdialysis during and after cytoreductive surgery combined with the HIPEC procedure. The model’s reproducibility was assessed by inserting dual catheters into the same tissues. In addition, catheters were positioned at varying depths in the stomach and rectum to assess tissue penetration.

## Methods

The study was conducted at the Institute of Clinical Medicine, Aarhus University Hospital, Aarhus, Denmark. Chemical analyses were performed at the Department of Biochemistry, University Hospital of Southern Jutland, Vejle. The study was approved by the Danish Animal Experiments Inspectorate (license no. 2017/15-0201-01184) and was conducted according to existing laws and institutional policies.

### Study Overview

**Fig. 1:**
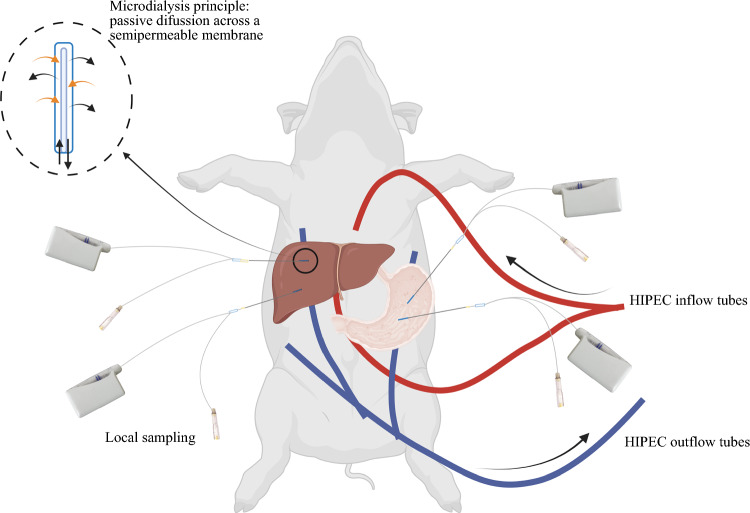
Overview of the HIPEC setup. Examples of placement of microdialysis catheters and HIPEC inflow and outflow tubes. The three outflow tubes were placed lateral for the liver, lateral for the spleen, and pelvis

In total, eight female pigs (Danish landrace breed, weight 58–62 kg, 4 months old) were included in the study. All animals underwent an open abdominal surgical procedure imitating cytoreductive surgery followed by 90 min of HIPEC. Cisplatin samples were obtained with microdialysis from the peritoneal lining of various abdominal tissues (Fig. 1, Table 1) during and after HIPEC (a total of 8 h of sampling). These locations were chosen to represent relevant peritoneal carcinomatosis locations. Cisplatin (Accord Healthcar B.V., Holland, Utrecht) was administered at a dosage of 100 mg/m^2^ over three fractions every 30 min.

**Table 1:** Catheter overview

Organ/tissue	Depth of microdialysis catheter placement	Number of microdialysis catheters
Liver	1 mm	2
Rectum	1–1.5 mm (superficial) and 2.5–3 mm (profound)	2
Stomach	1–2 mm (superficial) and 4–5 mm (profound)	2
Peritoneum	1 mm	3

^1^Placement of microdialysis catheters in the abdominal tissues, depths were verified by ultrasound. The two liver catheters were placed in similar locations 2 cm apart. Peritoneum 1 was placed at the peritoneal lining of the upper right abdominal wall, peritoneum 2 was placed at the peritoneal lining of the lower left abdominal wall, and peritoneum 3 was placed at the peritoneal lining of the upper left abdominal wall

### Microdialysis

This study utilized microdialysis equipment from M Dialysis AB (Stockholm, Sweden). The microdialysis system consisted of type 63 microdialysis catheters (membrane length, 30 mm; molecular cutoff, 20 kilodaltons) and CMA 107 precision pumps containing a perfusion fluid of 0.9% sodium chloride (NaCl) and continuously producing a flow rate of 1 µL/min. The placement of the 63 microdialysis catheters was performed using the SI-2 splitable introducer equipped with an integrated needle from M Dialysis AB (Stockholm, Sweden).

Microdialysis operates through diffusion across a semipermeable membrane at the catheter tip. The catheter is perfused with a solution, and substances below a certain size diffuse across the membrane, ensuring that only unbound drug concentrations are sampled. Given the constant perfusion of the microdialysis system, total equilibrium will never occur between the inside of the membrane and the surrounding tissue. Therefore, the dialysate drug concentration represents a fraction of the actual tissue concentration, known as relative recovery. In this study, the retrodialysis-by-drug method was used to calculate relative recovery^18^ using the equation

$$\text{Relative recovery}=100\times \left(1-\frac{{C}_{\text{dialysate}}}{{C}_{\text{perfusate}}}\right)$$

Here, *C*_dialysate_ is the cisplatin concentration in the dialysate (µg/mL), and *C*_perfusate_ is the cisplatin concentration in the perfusate (µg/mL). Absolute tissue concentration of free cisplatin (*C*_tissue_) was calculated using the following equation:

$${C}_{\text{tissue}}=100\cdot \frac{{C}_{\text{dialysate}}}{\text{RR}}$$

### Anesthesia and Surgical Procedures

During the experimental procedure, the pigs were kept under general anesthesia with continuous infusion of propofol (350 mg/h, Fresenius Kabi, Bad Homburg, Germany) and fentanyl (1.0–1.25 mg/h, B. Braun, Melsuneng, Germany). Arterial pH was kept within a range of 7.3–7.5. Rectal core temperature was kept within 36.5–39.0 °C. At the termination of the experiment, all pigs were euthanized by an overdose of intravenously administered pentobarbital.

With the animal in supine position, a surgical procedure imitating cytoreductive surgery was performed: through a midline abdominal incision from the xiphoid process to the symphysis, the ovaries and uterus were presented, and bilateral salpingo-oophorectomy and hysterectomy were performed. Total omentectomy was performed together with peritoneal stripping of 10 cm × 10 cm of the peritoneum from the left anterior abdominal wall. Following the surgical procedure, microdialysis catheters were placed in various abdominal tissues (Fig. 1, Table 1). The SI-2 splitable introducer was inserted into the tissue to the desired depth, a position that was subsequently verified using ultrasound imaging. Following the withdrawal of the needle contained within the introducer, a microdialysis catheter was carefully threaded through the lumen of the introducer. This meticulous positioning ensured that the catheter membrane was adequately situated within the tissue, parallel to the surface, thereby mitigating the risk of measurement contamination from intraabdominal fluids. The introducer was subsequently split and removed, resulting in the membrane remaining securely in place at a distance of 40 mm from the point of entry of the splitable introducer. To prevent potential dislocation of the catheters, they were externally secured to the tissue surface using sutures. In the rectum and the anterior wall of the stomach, catheters were placed at two depths (superficial and profound), as verified by ultrasound. After catheter placement, a 30-min tissue equilibration period was allowed.

### HIPEC Procedure

Initially, 4 L of 0.9% NaCl solution was heated and used as a carrier solution with 1 L as a reservoir in the pump system (Performer HT^®^ system from RAND, Medolla, Italy). A carrier solution volume of 4 L was chosen to imitate standard clinical regimes.^19^ Using the open technique, two inflow tubes were placed within the abdominal cavity, and three outflow tubes with attached temperature probes were placed via incisions in the abdominal wall near the liver, spleen, and pelvis. In addition, temperature probes were connected to the main inflow and outflow tubes. When the intraabdominal temperature was stable at a minimum of 41 °C, a total dose of 100 mg/m^2^ cisplatin was split into three and administered at 30-min intervals.^2,20^ The first dose, corresponding to 50% of the total dose, was administered at a defined time of 0. The second and third doses, each corresponding to 25% of the total dose, were administered after 30 min and 60 min. The total HIPEC treatment time was 90 min.

The body surface area for each pig was calculated with the following equation^21^:

$$\text{SA}=0.0970\cdot {W}^{0.633}$$

where SA is the pig’s body surface area (m^2^) and *W* is the body weight (kg). On the basis of the calculation, pigs weighing 60 kg had an SA of 1.295 m^2^. The temperature within the abdominal cavity was kept between 40 and 42 °C during the HIPEC procedure in all temperature probes. The abdominal cavity was drained entirely immediately after the HIPEC procedure.

### Sampling of Cisplatin Dialysates

**Fig. 2:**
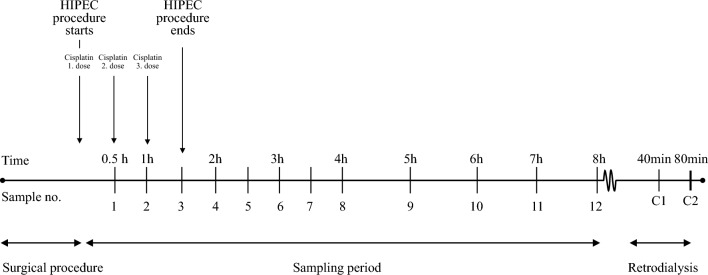
Illustration of sampling and procedure timeline. Time is shown above the timeline, and sample numbers are displayed under it. The time starts from the initiation of the HIPEC procedure, and the time for administering the three cisplatin doses is shown in the fig. Calibration samples from the calibration period (retrodialysis) are named C1 and C2

Dialysate samples were collected for 8 h, with the initiation of HIPEC indicating time zero. The sampling intervals were every 30 min from 0 to 240 min and every 60 min from 240 to 480 min (Fig. 2). A total of 12 samples were collected from each catheter. Catheters displaced or found outside the intended target tissue at autopsy were excluded from analysis.

### Stabilizing of Dialysates for Cisplatin Analysis

Optimal stabilizing of cisplatin samples has previously been established.^22^ The optimal stabilizing solution used for 10 µL of dialysate consisted of 400 µL of a premix containing 170 parts methanol, 10 parts of NaCl 18% in water, and 15 parts dichloro(ethylenediamine)platinum(II) 20 µg/L in water (12.8% vol/vol). Immediately upon collection, the dialysates were pipetted and stabilized with this solution.

### Ultra-Performance Liquid Chromatography-Tandem Mass Spectrometry (UPLC-MS/MS)

Cisplatin dialysate concentrations were analyzed by UPLC-MS/MS. A detailed description can be found elsewhere.^22^ The lower limit of quantification was 0.030 µg/mL, with an intermediary precision of around 20%.

### Pharmacokinetic Model and Statistical Analysis

Using Stata (version 18.0), the pharmacokinetic parameters were described by a two-compartment model with a zero-order distribution from the central compartment to the peripheral compartment and a first-order elimination from the peripheral compartment. The advantage of this approach is that more precise values of the pharmacokinetic parameters are obtained, provided that the nonlinear regression model describes the data adequately, which was deemed to be the case here. The initial drug concentration in the peripheral tissue was set to 0. The rate constants and the initial concentration in the central compartment were estimated using a nonlinear mixed effects model for each abdominal tissue. Pharmacokinetic parameter estimates, the area under the curve from time zero to the time of the last sample collection (AUC_0–last_), time to peak drug concentration (*T*_max_), peak drug concentration (*C*_max_), and half-life (*T*_1/2_) were calculated for each animal and each tissue/catheter.

Differences between tissues are presented as ratios of estimated medians (back-transformed differences on the log scale) with 95% confidence intervals. The significance level was set at 5%.

To illustrate inter-catheter variation within the same tissue, each tissue’s limits of agreement (LOA) were calculated using a linear mixed model with a random animal effect. The LOA is a 95% prediction interval for the difference between two catheters in the same tissue in the same animal, so 95% of such observed differences will lie within ± LOA. LOA are presented as median ratio prediction intervals.

## Results

All eight pigs completed the study. Malfunction or displacement of catheters outside the target tissue was observed in 19 of the 62 catheters. Mean relative recoveries for the individual tissue compartments are presented in Table 2.

**Table 2:** RRs of tissue compartments

Compartment	Mean (SD) relative recovery
Liver 1	86.4% (10.6)
Liver 2	89.6% (4.0)
Rectum superficial	85.4% (22.2)
Rectum profound	70.3% (8.8)
Stomach superficial	87.0% (11.4)
Stomach profound	91.0% (4.9)
Peritoneum 1	73.0% (9.2)
Peritoneum 2	75.5% (10.9)
Peritoneum 3	73.9% (18.0)

Relative recoveries are reported as mean (SD) for all compartments

**Fig. 3:**
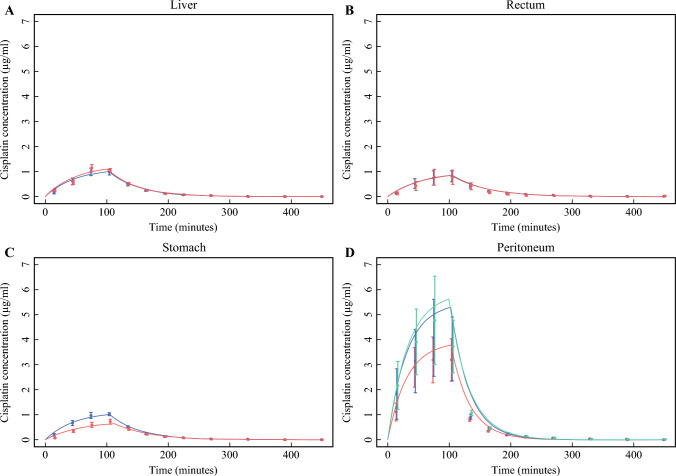
Time–concentration curves for each abdominal tissue with standard errors (SE). Fitted curves from the nonlinear mixed effects model are superimposed. **A** Red and blue lines depict liver compartments 1 and 2, respectively, **B** the red curve signifies the profound compartment, the black curve corresponds to the superficial compartment for the rectum, and **C** represents the stomach, **D** peritoneal compartments 1, 2, and 3 are denoted by red, blue, and green lines, respectively

Figure 3 depicts the mean time–concentration curves for each abdominal tissue compartment. The lowest and highest median *C*_max_ values within the investigated tissues were found in the profound rectum (0.45 µg/mL) and the peritoneum (3.11 µg/mL), respectively. No statistically significant differences in cisplatin *C*_max_ were found between the abdominal compartments, except for the peritoneal tissue where *C*_max_ was significantly higher than in all of the other abdominal organ tissues. Similarly, no statistically significant differences in cisplatin AUC_0–last_ were found between the abdominal compartments, except for the peritoneal tissue where AUC_0–last_ was significantly higher than in all of the other abdominal tissues. The smallest median ratio was found for peritoneum 1/liver 2; 2.27, (95% CI 1.13; 4.56, *P* = 0.022), and the most significant median ratio was found for peritoneum 3/rectum profound; 6.15 (95% CI 2.84; 13.33, *P* = 0.000).

*T*_max_ was comparable between most compartments, ranging from 101 to 124 min, and *T*_1/2_ was comparable between all compartments, ranging from 16 to 41 min, except for the peritoneal tissue, which was significantly lower than all other compartments and the profound stomach compartment, which was higher than the superficial stomach and the liver compartments (Table 3).

**Table 3:** Pharmacokinetic data for cisplatin during HIPEC procedure

Compartment	*n*	*AUC*_0–last_	*T*_1/2_ (min)	*C*_max_ (µg/mL)	*T*_max_ (min)
Liver 1	7	115.0 (73.3; 180.4)	33.7 (30.8; 36.8)	1.0 (0.6; 1.6)	103.5 (102.0; 116.5)
Liver 2	5	125.0 (74.4; 212.7)	32.6 (29.3; 36.3)	1.1 (0.6; 2.0)	100.6 (96.8; 113.2)
Rectum superficial	4	104.3 (61.3; 177.5)	36.9 (33.2; 41.1)	0.8 (0.4; 1.4)	124.4 (110.3; 140.4)
Rectum profound	4	58.0 (32.0; 105.1)	35.5 (31.4; 40.0)	0.5 (0.2; 0.9)	115.5 (101.0; 132.2)
Stomach superficial	7	118.3 (75.5; 185.6)	34.5 (31.5; 37.8)	1.0 (0.6; 1.7)	102.9 (92.9; 114.0)
Stomach profound	6	80.6 (49.6; 131.1)	41.0 (37.2; 45.2)	0.6 (0.4; 1.1)	108.0 (96.8; 120.6)
Peritoneum 1	8	284.1 (186.4; 432.8)	21.3 (19.5; 23.1)	2.6 (1.6; 4.2)	104.8 (95.3; 115.3)
Peritoneum 2	6	306.4 (188.5; 498.1)	15.9 (14.4; 17.5)	2.7 (1.6; 4.6)	114.6 (102.6; 127.9)
Peritoneum 3	6	357.1 (227.6; 559.9)	21.6 (19.5; 23.4)	3.1 (1.9; 5.1)	110.6 (99.8; 122.4)

Concentrations are given as medians; the 95% confidence intervals (95% CI) for all compartments are given in parentheses

Higher cisplatin concentrations were found in superficial tissues; however, the difference was not statistically significant. For the stomach, higher median AUC_0–last_ and *C*_max_ values were found for the superficial placement compared with the profound placement [AUC_0–last_ 118.34 vs. 80.64 (ratio 0.68, 95% CI 0.35–1.34, *P* = 0.26) and *C*_max_ 1.00 vs. 0.63 µg/mL (ratio 0.63, 95% CI 0.30–1.34, *P* = 0.22)]. For the rectum, the median AUC_0–last_ and *C*_max_ values for the superficial placement were also higher than the profound placement (AUC_0–last_ 104.27 vs. 58.00 (ratio 0.56, 95% CI 0.24–1.27, *P* = 0.16), and *C*_max_ 0.75 vs. 0.45 µg/mL (ratio 0.59, 95% CI 0.24–1.49, *P* = 0.26)).

**Fig. 4:**
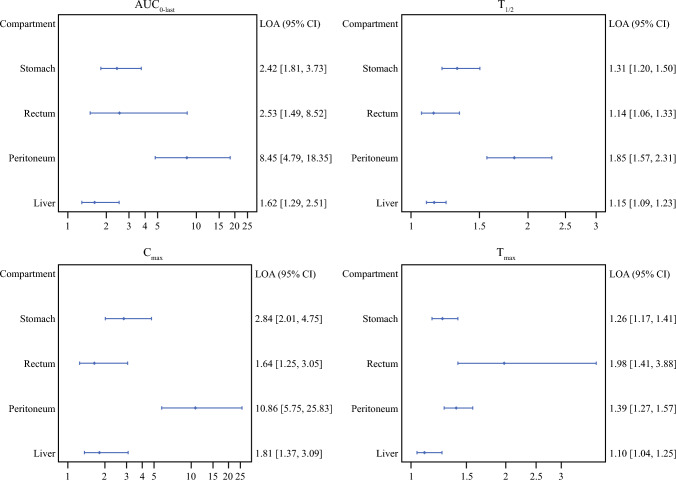
LOA values illustrating the variation between catheters in the same tissue. Medians for two catheters in the same tissue in the same animal are within a factor LOA of each other (with 95% confidence intervals)

For AUC_0–last_, *C*_max_, and *T*_1/2_, notable inter-catheter variation within the same tissue was seen, especially in the peritoneum and rectum (Fig. 4). The smallest variation was observed between the two liver catheters.

## Discussion

### Summary of Main Results

This porcine model effectively evaluated cisplatin concentrations in abdominal organs during HIPEC treatment. We found comparable cisplatin concentrations in the stomach, rectum, and liver, with higher concentrations in the peritoneal lining of the abdominal wall. Cisplatin remained detectable in tissues for at least 6 h post-HIPEC.

Concentrations tended to decrease with tissue depth, suggesting abdominal rather than systemic circulation as the source. Further studies on cisplatin concentrations after intravenous administration are needed to identify the contribution from systemic re-circulation.

### Results in the Context of Published Literature

Limited data exist on the target tissue pharmacokinetics of intraperitoneal cisplatin during and after HIPEC. One rat study applying cytoreductive surgery with HIPEC using an abdominal cisplatin injection reported a liver *C*_max_ nearly double that observed in our study.^23^ Another HIPEC porcine study reported a peritoneal cisplatin *C*_max_ of 18.8 µg/mL, significantly higher than the concentrations found in the present study (2.6 µg/mL).^12^ Data are, however, hard to compare owing to fundamental differences in study set-up and quantification methods. These studies utilized the biopsy method, and samples were quantified by inductively coupled plasma-mass spectrometry (ICP-MS), which measures total platin (bound + unbound + metabolites of cisplatin). As the unbound drug is directly correlated to therapeutic efficacy, we aimed to sample only the free cisplatin.^17^ Moreover, the HIPEC porcine study applied a cisplatin dose of 70 mg/m^2^ using the closed method, and the target temperature was 43 °C.^12^

### Strengths and Weaknesses

This is the first study to apply microdialysis to assess in vivo cisplatin abdominal tissue concentrations during and after HIPEC in a porcine model. We used pigs weighing between 58 and 62 kg to resemble a human-sized abdominal cavity. Although pigs are similar to humans in physiology and anatomy, there are several essential aspects to consider when evaluating the translational potential of the present data.^24^ To mimic the clinical setting, we performed a cytoreductive procedure similar to that undergone by patients with ovarian cancer before the HIPEC procedure, although it was not as extensive as most cases are. The pigs were young and healthy, i.e., they did not have peritoneal carcinomatosis. Tumor tissue may have characteristics that affect the microenvironment and penetration of chemotherapeutic compounds into the cells, which could not be accounted for in this large animal model.^25,26^ While this study does not involve tumor-bearing tissues, it provides a critical foundation for understanding the basic pharmacokinetics of cisplatin in a controlled HIPEC setting. Future studies should aim to bridge these findings to tumor-bearing models or clinical trials to evaluate the therapeutic implications for patients with peritoneal malignancies.

The sample size of eight pigs was guided by ethical considerations rooted in the principles of the 3Rs (replacement, reduction, refinement) and practical limitations that align with established standards for preclinical pharmacokinetic studies in large animal models.^27^ While this may represent a limitation in terms of generalizability, microdialysis is significantly advantaged by utilizing dense microdialysis sampling (collection of 12 samples per catheter) to yield robust pharmacokinetic data for each target tissue analyzed.

Despite meticulous efforts to secure catheters within the abdominal tissue, 19 catheters were displaced during the experiments. As this was our first study with this new HIPEC model, we did not anticipate the challenges of catheter displacement. We suspect this to be caused by fluid movements and intestinal peristalsis. Thus, from our perspective, this reflects a surgical and methodological learning curve rather than inadequacy of the model and microdialysis, which we have successfully improved for upcoming studies.

We assessed method reproducibility by placing more catheters within the same organ and depth. We found a considerable LOA value for the peritoneal compartments, which may be explained by the fact that they were placed in three different quadrants of the abdomen. Also, HIPEC is a local treatment, and although this intuitively may be expected to provide low LOA values, other local treatment forms (e.g., antibiotics) have proven to provide varying concentrations, presumably owing to differences in the drug dilution potential influenced by the target tissue density and architecture.^28,29^ Moreover, during the HIPEC procedure, it became evident that the temperatures of the chemotherapeutic solution were lower in the pelvis compared with the rest of the abdomen. This indication of heterogeneous temperature distribution may also apply to the chemotherapeutic solution, potentially accounting for some of the present variation.

### Implications for Practice and Future Research

An in-depth understanding of drug concentrations within target tissues is imperative for optimizing the antineoplastic efficacy of different chemotherapeutics used in HIPEC procedures. The establishment of this porcine model provides a crucial foundation for forthcoming investigations, offering a novel and necessary platform for investigating and optimizing HIPEC procedures such as treatment temperature regulation, chemotherapeutic drug dosage, and procedure duration. Examples of this include the comparative analysis of HIPEC and normothermic intraperitoneal chemotherapy (NIPEC), the investigation of various chemotherapeutic agents, including localized therapeutic modalities such as pressurized intraperitoneal aerosol chemotherapy (PIPAC), and the evaluation of perfusion and dosage parameters to optimize tissue concentrations and exposure. These initiatives can potentially refine HIPEC protocols, thereby enhancing therapeutic outcomes.

## Conclusions

The application of microdialysis proved feasible for evaluating local cisplatin concentrations in abdominal tissues during and following HIPEC treatment in a large porcine model. We found a comparable distribution pattern of cisplatin throughout the investigated abdominal organ tissues (the stomach, rectum, and liver) but higher concentrations in the peritoneal lining of the abdominal wall. Cisplatin was detectable in the target tissues for a minimum of 6 h after the completion of the HIPEC procedure and exhibited a penetration depth of at least 4 mm.

## Supplementary Information

Supplementary file 1
