# Supplementary material for: Dynamic Assessment of Local Abdominal Tissue Concentrations of Cisplatin During a HIPEC Procedure: Insights from a Porcine Model
Source: Ann Surg Oncol. 2025 Feb 12;32(5):3804–13. doi: 10.1245/s10434-025-17000-w (PMC11976807; doi:10.1245/s10434-025-17000-w)

# Supplemental materials

**Supplemental materials A-D Time concentration curves for abdominal compartments:**

*Figure A-D shows the time-concentration curves for each pig (pig.catheter in the panel headers) each graph corresponding to a specific compartment in the abdominal tissue. Superimposed are the fitted curves from the non-linear mixed effects model revealing a good description of the data by the compartment model.*

### A. Liver compartments


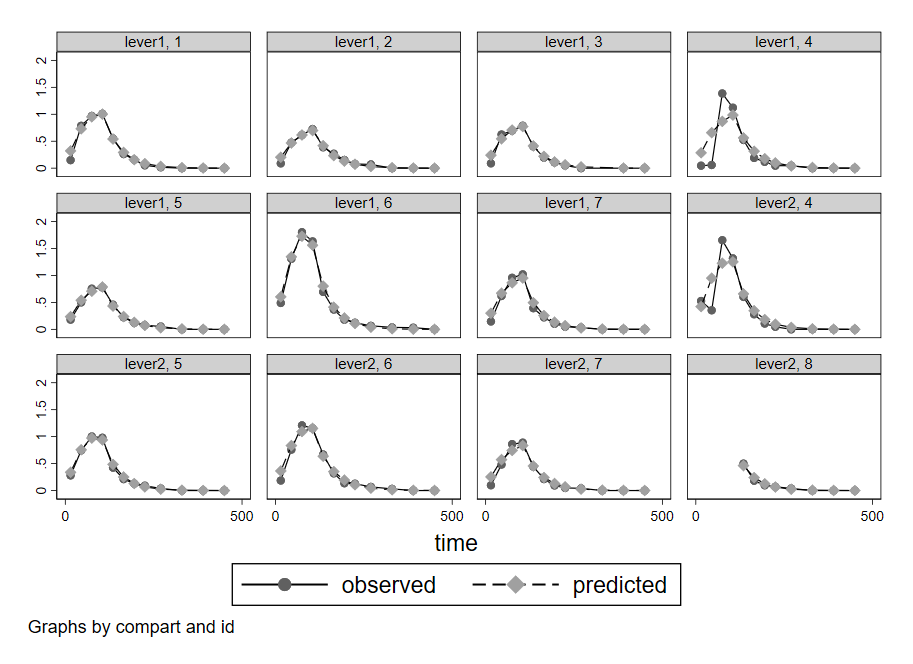


### IIB. Stomach compartments


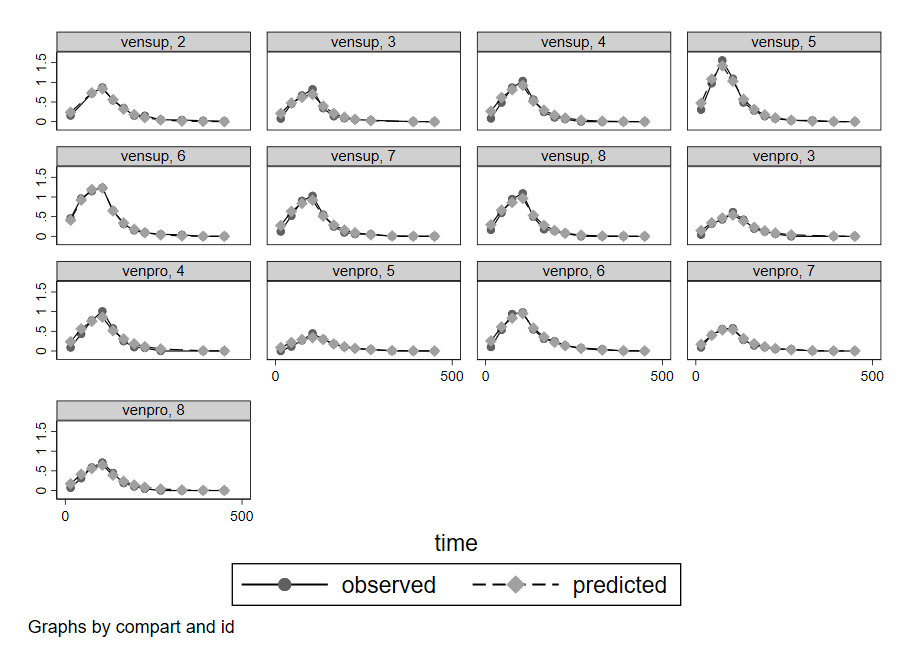


### IIC. Rectal compartments


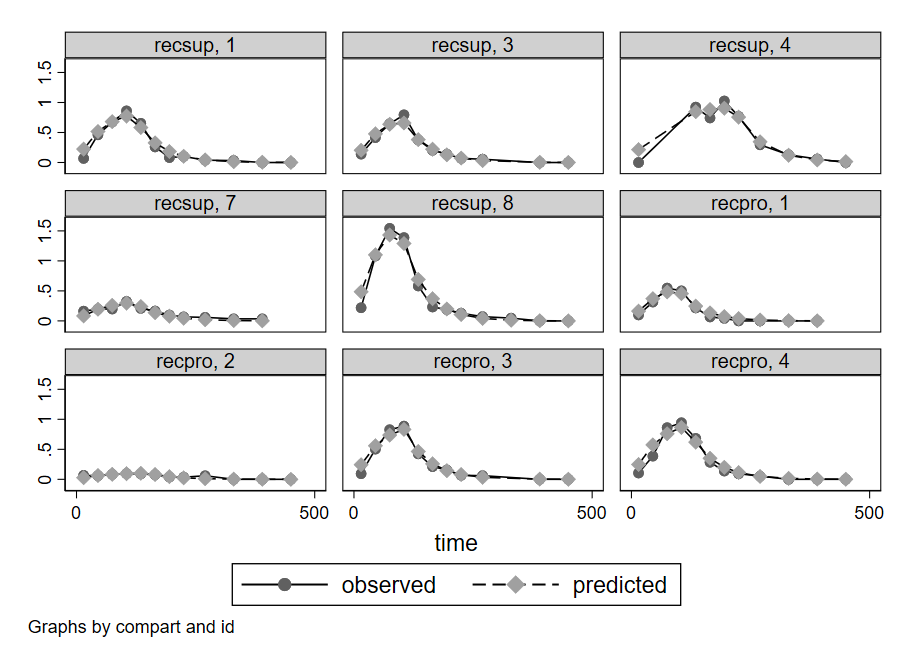


### II.D Peritoneal compartment


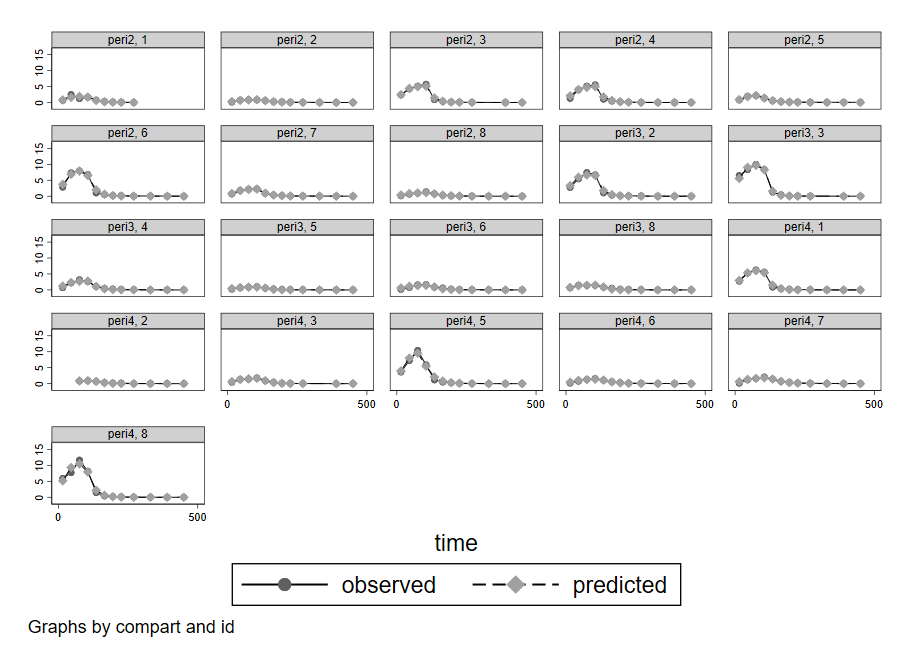

Supplement: Supplementary file 1 [file 10434_2025_17000_MOESM1_ESM.docx]
